# Postoperative Outcomes Following Cholecystectomy: A Nationwide Analysis

**DOI:** 10.7759/cureus.83974

**Published:** 2025-05-12

**Authors:** Emma Vella, Maria Borg, Svetlana D Brincat, Christian Camenzuli

**Affiliations:** 1 General Surgery, Mater Dei Hospital, Msida, MLT; 2 Family Medicine, Primary Health Care, Floriana, MLT

**Keywords:** cholecystectomy, cholecystitis, gallstones, post-operative complications, readmission rate

## Abstract

Introduction

Laparoscopic cholecystectomy has revolutionized the surgical approach to gallbladder removal and has since then become the gold standard. Despite the significant benefits of laparoscopic cholecystectomy, complications requiring hospital readmission remain a cause for morbidity. The nationwide audit aims to assess the 30-day readmission rate following cholecystectomy in Malta and compare these findings to guidelines recommending a readmission rate of less than 10%.

Method

All patients, male and female, who underwent a cholecystectomy in the year of 2023 were identified from the hospital operating theater records. After compiling the patient list and obtaining ethical approval, hospital records, including electronic discharge summaries and radiological investigations, were reviewed to collect data on demographics, indication for cholecystectomy, and cause for readmission.

Results

In 2023, a total of 288 patients underwent cholecystectomy at Mater Dei Hospital. Of these, 168 (58.3%) patients were female and 120 (41.7%) were male, with the majority falling within the 50-59 age range. Most surgeries, n = 282 (97.9%), were elective, with only six patients requiring emergency intervention. Cholecystectomy was performed in the majority of cases as laparoscopy n = 279 (96.9%), one surgery was performed as an open procedure (<1%), and eight cases (2.8%) were converted from laparoscopic to open surgery.

The most common indication for cholecystectomy was cholecystitis (n=138, 47.9%), cholelithiasis (n=89, 30.9%), and pancreatitis (n=45, 15.6%). Two patients had cholecystectomy for malignancy (n = 2, 0.69%). The remaining patients had surgery secondary to porcelain gallbladder (n = 2), gallbladder polyps (n = 7), rupture (n = 1), adenomyosis and dysmotility (n = 4), collectively making up 4.86%. Preoperative imaging with magnetic resonance cholangiopancreatography (MRCP)/endoscopic retrograde cholangiopancreatography (ERCP) was conducted in 175 patients (60.7%), while 113 patients (39.2%) did not undergo any form of imaging prior to surgery.

Regarding postoperative outcomes, 84 patients (29.1%) were discharged on the same day as their procedure, with the majority, n = 128 (44.4%), discharged the following day. N = 50 (17.36%) patients stayed in hospital for 2-5 days, n = 21 patients stayed in hospital for 6-10 days (7.30%). Only n = 5 (1.74%) patients stayed in the hospital for > 10 days.

In total, 13 patients (4.5%) were readmitted, with the most common reasons being surgical complications, n = 9 (69.2%). Other causes of readmission included medical complications, which account for n = 4 (30.8%) of the readmitted cohort.

Conclusion

The 30-day readmission rate stood at 4.5%. This rate should be considered in light of the fact that the majority of patients were discharged just one day after surgery.

## Introduction

Cholecystectomy is one of the most commonly performed surgical procedures worldwide, primarily indicated for the treatment of gallbladder-related conditions, including cholelithiasis and acute cholecystitis. The implementation of laparoscopic cholecystectomy has transformed surgical practice, establishing itself as the preferred approach due to its numerous advantages over traditional open surgery [1]. This minimally invasive technique not only reduces recovery times and postoperative pain but also shortens hospital stays, significantly enhancing patient satisfaction and quality of life [2].

Despite these considerable benefits, discrepancies in clinical outcomes persist, with some patients experiencing complications that lead to readmissions following surgery. Factors contributing to these variations may include patient-specific characteristics such as age and underlying health conditions, as well as surgical variables like the experience of the operating surgeon and unforeseen intraoperative complications. As the complexity of gallbladder disease evolves, understanding the reasons behind readmissions becomes crucial for improving overall patient care [3].

Aim

This audit aims to evaluate the 30-day readmission rate following laparoscopic cholecystectomy at Mater Dei Hospital, the only national hospital in Malta. In addition to determining the readmission rate, the study aimed to investigate the underlying causes of these occurrences, identifying trends that may inform clinical practice. Furthermore, the findings will be compared to international benchmarks, specifically those outlined by the Royal College of Surgeons of England Commission’s guideline, which recommends a readmission rate of less than 10% [4]. The CholeS UK study and Royal College of Surgeons guidelines were selected due to their wide recognition, robust methodology, and relevance to laparoscopic cholecystectomy practice across the UK and similar healthcare systems such as Mater Dei Hospital.

## Materials and methods

Study design

This retrospective study included all patients who underwent cholecystectomy in the year 2023 at Mater Dei Hospital in Malta. Patients were identified through hospital list records when the surgery type was coded as “cholecystectomy”. Electronic discharge summaries and iSOFT Clinical Manager® were used to identify patient demographics, discharge, indication for surgery, date of readmission, and complications. Radiological investigations through Picture Archiving and Communication System (PACS) were also used to gather data on postoperative complications. Data was inputted and analyzed manually in Microsoft Excel®.

Population size

Two hundred eighty-eight patients underwent laparoscopic and open cholecystectomy in Mater Dei Hospital during the year 2023.

Inclusion criteria

All patients, male and female, Maltese or foreign, who underwent cholecystectomy in the year 2023 at Mater Dei Hospital, regardless of indication.

Exclusion criteria

Cholecystectomy performed in Maltese private hospitals or centers, readmission to hospital beyond 30 days post-operation.

Ethics

This study was approved by the Ethics Board at Mater Dei Hospital, Malta. Due to the retrospective nature of the audit, the requirement for informed consent was waived. All information was anonymized and handled in accordance with hospital guidelines and the General Data Protection Regulation (GDPR).

## Results

**Figure 1:**
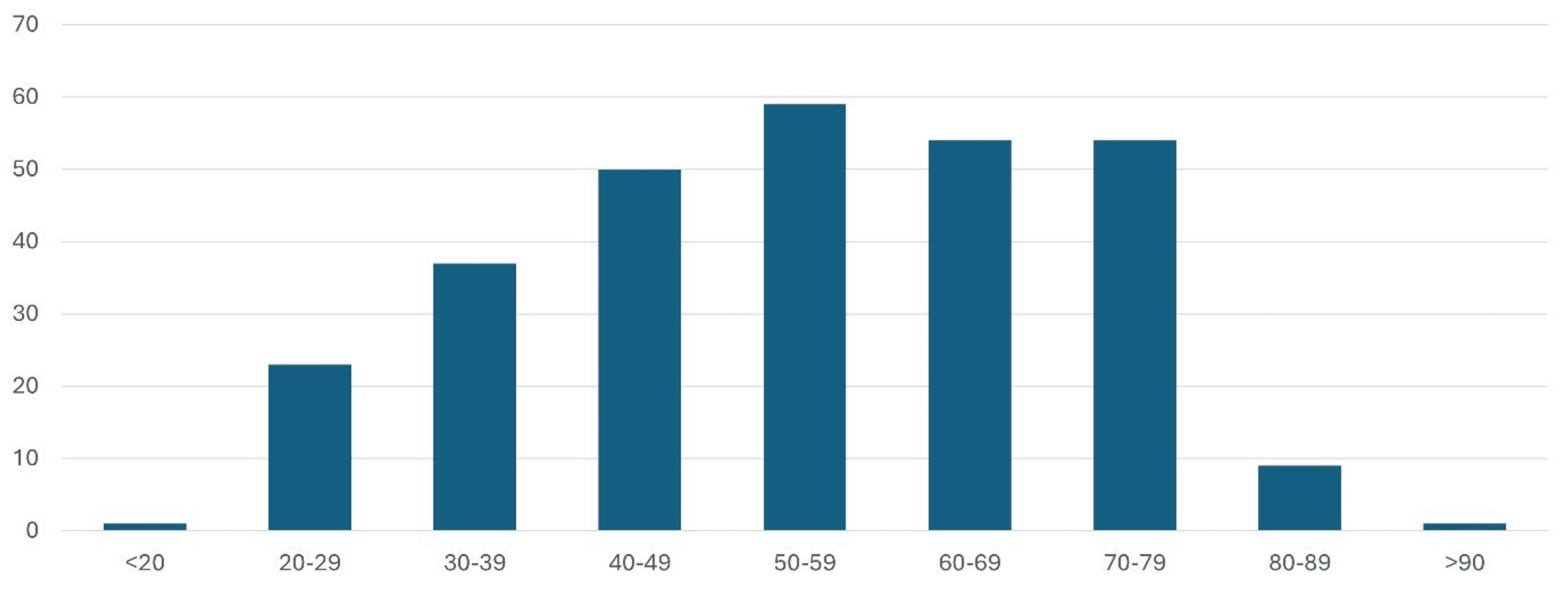
Bar chart showing the mean age of patients undergoing cholecystectomy

Two hundred eighty-eight patients underwent cholecystectomy at the national hospital in Malta in 2023. One hundred twenty patients (41.6%) were men, while 168 patients (58.4%) were women. The majority of patients (20.4%, n=59) were within the 50-59-year age range (Figure 1). Two hundred eighty-two cases (97.9%) of the cholecystectomies were performed electively, with only six being emergency cholecystectomies. Of all the cholecystectomies, 279 cases (96.9%) were laparoscopic, one case was open, while 8 cases were planned laparoscopic but converted to open cholecystectomy. All cases (n=6) performed in an emergency setting were performed laparoscopically.

**Figure 2:**
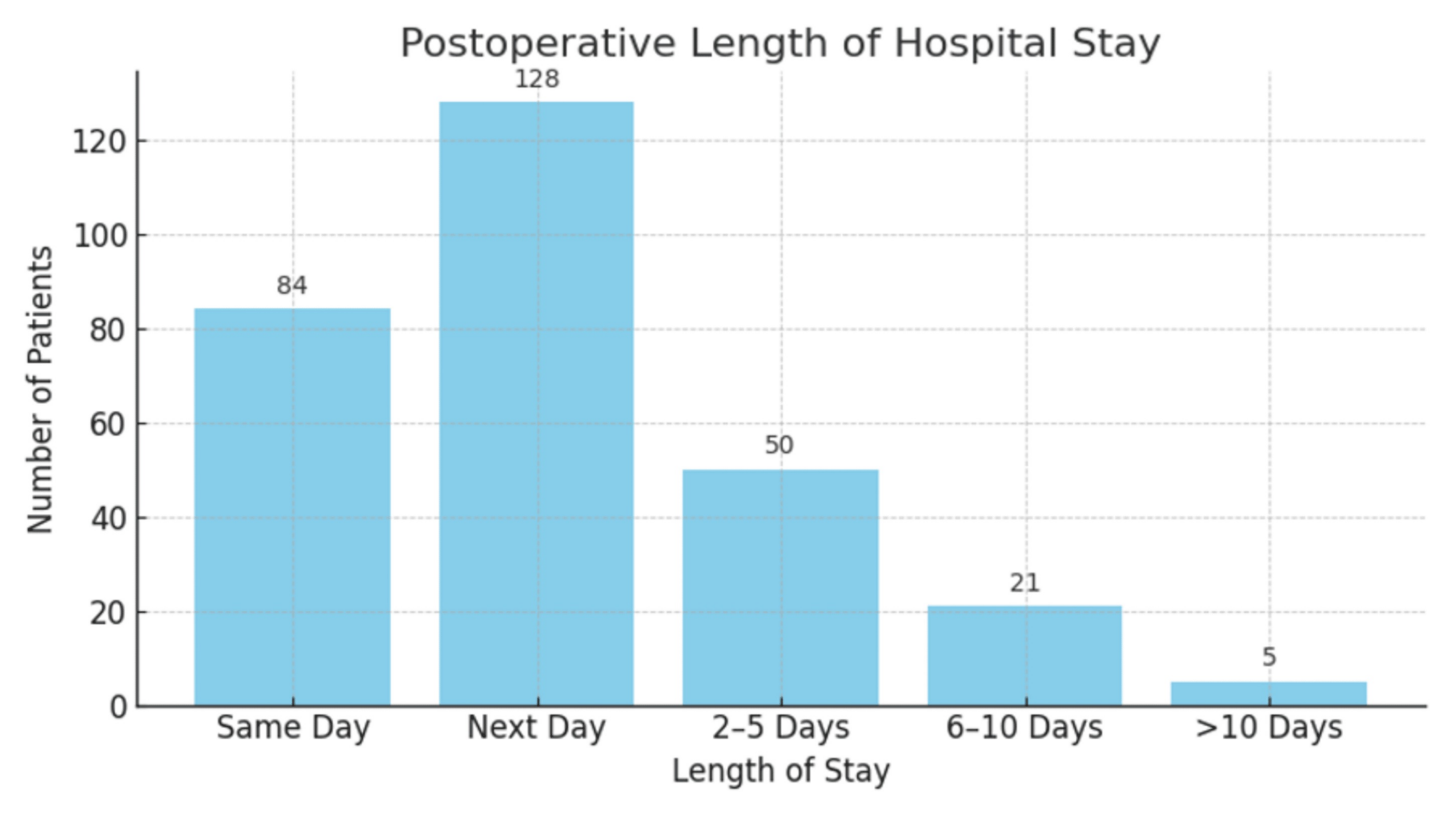
Bar chart showing postoperative length of stay

Regarding postoperative outcomes, 84 patients (29.1%) were discharged on the same day as their procedure, with the majority, n = 128 (44.4%), discharged the following day. N = 50 (17.36%) patients stayed in hospital for 2-5 days, n = 21 patients stayed in hospital for 6-10 days (7.30%). Only n = 5 (1.74%) patients stayed in the hospital for > 10 days. Of the six patients who underwent emergency cholecystectomy, the average length of stay was 7 days (Figure 2). 

**Figure 3:**
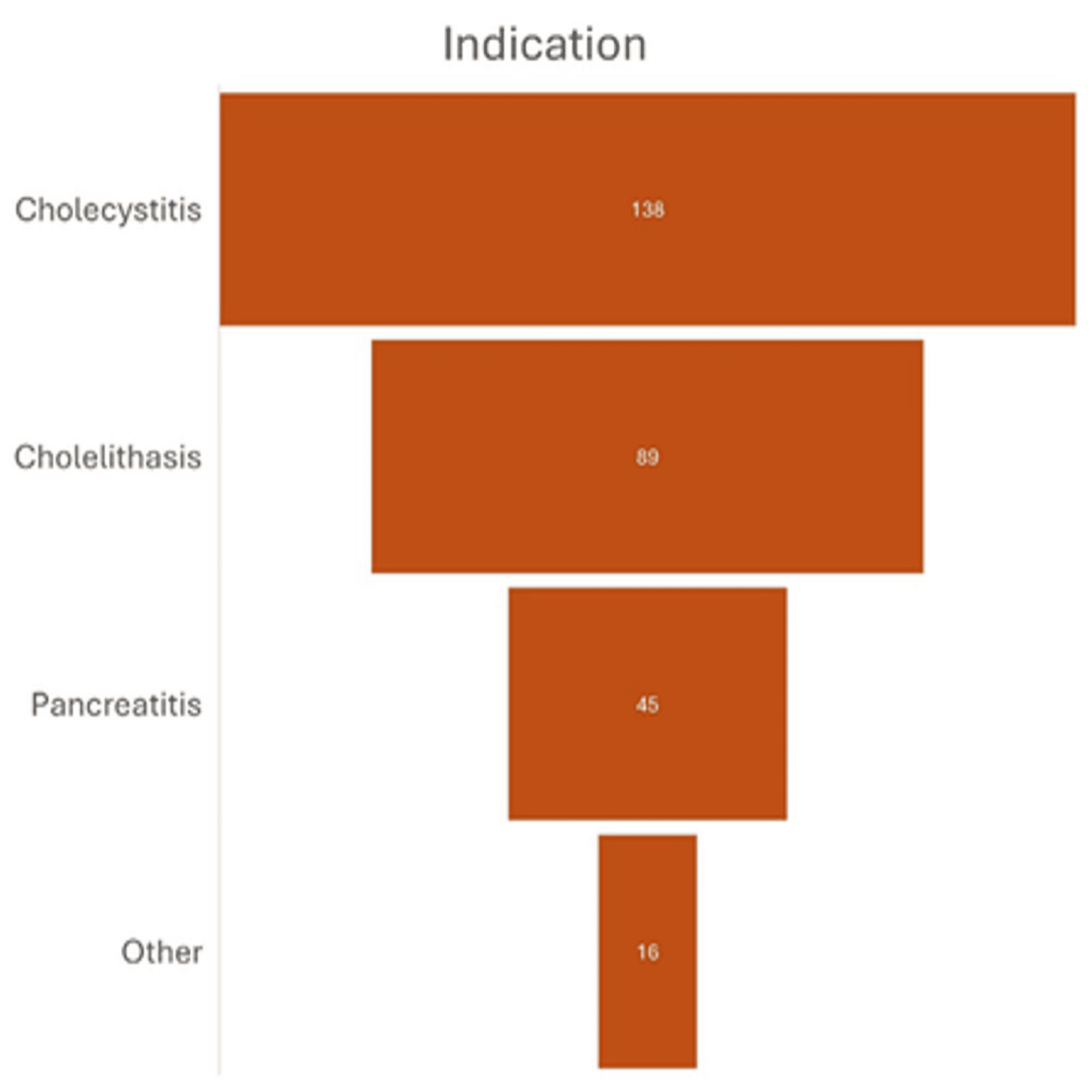
Bar chart showing the indications for cholecystectomy

The most common indication for cholecystectomy was cholecystitis (n=138, 47.9%), cholelithiasis (n=89, 30.9%), and pancreatitis (n=45, 15.6%). Two patients had cholecystectomy for malignancy (n = 2, 0.69%). The remaining patients had surgery secondary to porcelain gallbladder (n = 2), gallbladder polyps (n = 7), rupture (n = 1), adenomyosis and dysmotility (n = 4), collectively making up 4.86% (Figure 3).

One hundred seventy-five patients (60.7%) were reported to have had a preoperative magnetic resonance cholangiopancreatography (MRCP) or endoscopic retrograde cholangiopancreatography (ERCP), and eight patients (2.7%) had intraoperative on-table cholangiogram. Of the six emergency cases, only two had preoperative MRCP. 

Of the 288 total patients, 6 underwent emergency cholecystectomy, 104 patients never had a previous related admission, and the remaining 178 patients had related previous admissions to the hospital. Four of the six emergency cholecystectomy patients had no previously related admissions to the hospital prior to surgery.

**Figure 4:**
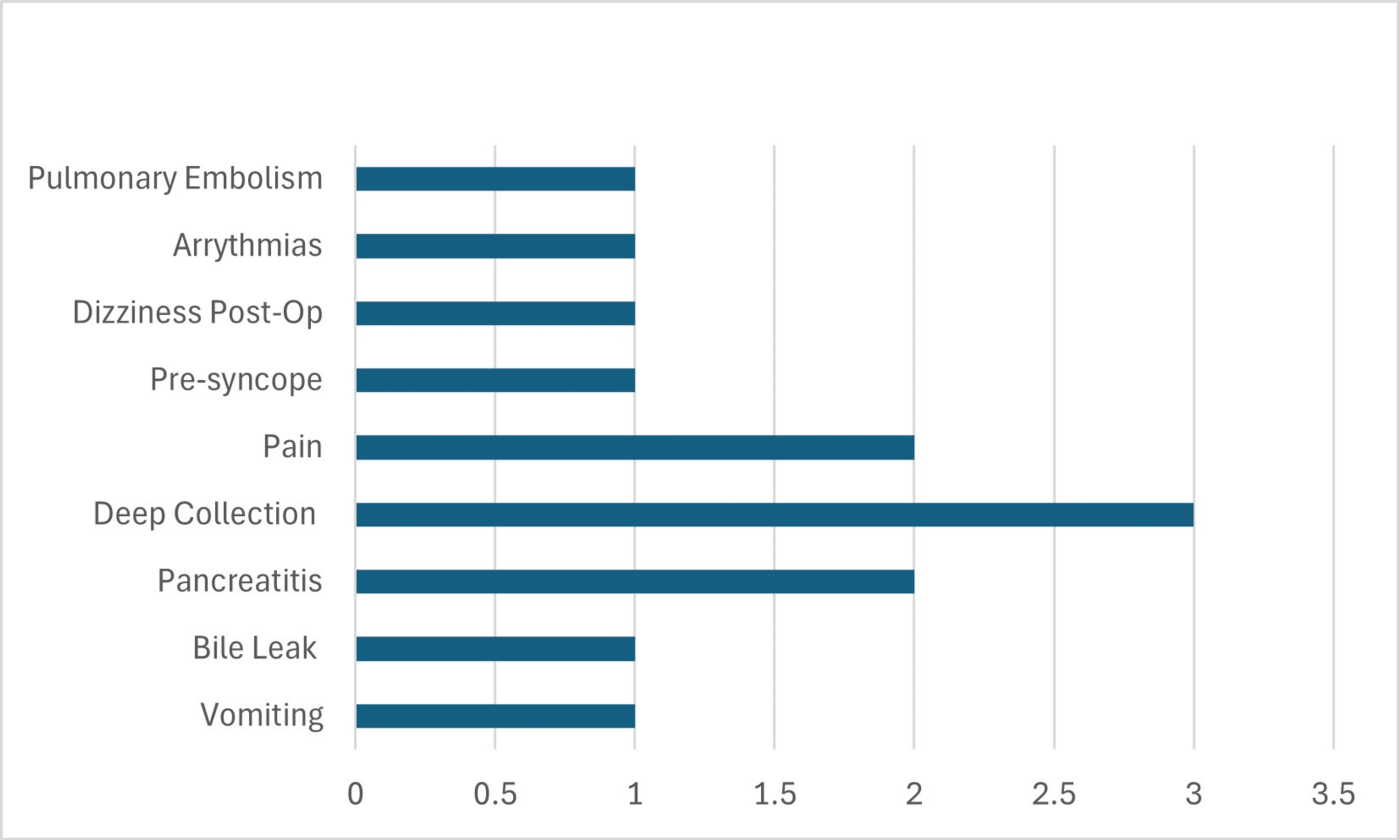
Bar chart showing readmissions

A total of 13 patients (4.5%) were readmitted to the hospital within 30 days of surgery (Figure 4). All readmitted patients underwent elective surgery. The most common surgical readmission was due to deep collections (n=3, 23%), followed by abdominal pain (n=2, 15.4%), bile leak (n=1, 7.7%), pancreatitis (n=2, 15.4%), and nausea and vomiting (n=1, 7.7%). Collectively, medical complaints (including dizziness, presyncope, arrhythmias, and pulmonary embolism) contributed to the rest of the readmissions (n=4, 30.8%). 

Thirty-eight patients experienced complications, including those who remained inpatient and those who were readmitted, accounting for 13.1% of the total 288-cohort. Of these, 24 patients experienced direct surgical complications, the most common being (in descending order) deep infective fluid collection (n=5), vomiting (n=4), urinary retention (n=4), abdominal pain (n=4), bile leaks (n=3), pancreatitis (n=2), perforation (n=1), and peri-arrest with eventual death (n=1). Fourteen patients experienced post-op medical complications with the commonest being (in descending order) lower respiratory tract infections (n=3), ECG changes post-op (n=2), thrombosis (n=2), presyncope (n=2) heart failure exacerbation (n=1), confusion (n=1), and other non-specific complaints (n=3). Only one mortality post-op was recorded.

None of the emergency cholecystectomy patients were readmitted within 30 days.

## Discussion

Laparoscopic cholecystectomy has become the gold standard procedure for the removal of a diseased, malignant, or painful gallbladder. The laparoscopic approach has replaced open cholecystectomy since the 1990s, with the open technique being used when the former fails or in cases of malignancy where more exposure is essential [5]. A minimally invasive approach is associated with overall better outcomes, less patient morbidity, and shorter hospital stays [6]. In our audit, the majority of cholecystectomies were performed using a laparoscopic approach and in an elective setting.

Readmission rates are a critical indicator of the quality of medical care. Patient readmissions contribute to increased costs, strain on healthcare resources, inconvenience, heightened morbidity due to prolonged stays, and even potential mortality. Factors influencing readmission rates after cholecystectomy include patient demographics, surgical complexity, underlying indications, and pre-existing health conditions [1].

The CholeS study, a large-scale national audit conducted in the UK, provided valuable insights into the 30-day readmission rates following laparoscopic cholecystectomy. According to the findings, the ideal 30-day readmission rate should be less than 10%, which aligns with national benchmarks aimed at maintaining high standards of postoperative care. The study highlighted that readmission rates are a key quality indicator, reflecting the overall effectiveness of surgical and postoperative management. The findings of this audit align with national CholeS guidelines, with this audit recording a readmission rate of 4.5%, meeting these standards. Evidence has demonstrated that day-surgery laparoscopic cholecystectomy is as safe as procedures involving an overnight stay, as earlier discharges do not inherently result in higher readmission rates [4]. Notably, all readmissions in this audit were associated with elective cholecystectomies, while none of the emergency cases required readmission.

Several factors may contribute to this outcome. Emergency cholecystectomies and cholecystectomies secondary to malignancy often receive more intensive postoperative monitoring and care, which helps to minimize complications that could lead to readmission. Additionally, these patients may remain in the hospital for a longer initial observation period, ensuring that any early complications are managed before discharge. These combined factors likely explain the absence of readmissions among patients who underwent emergency cholecystectomies in this audit.

The CholeS study reported an overall complication rate of approximately 10.8% for laparoscopic cholecystectomy, encompassing a spectrum of issues from minor complications to more severe outcomes, such as bile leaks, infections, bleeding, and bile duct injuries. In comparison, our audit found a slightly higher complication rate of 13.1%, which is higher than the CholeS benchmark [4]. Possible reasons why this might be the case include the increasingly older cohort, the fact that laparoscopy is not only performed by hepatobiliary surgeons (but all general surgeons have such cases), and that hot laparoscopic cholecystectomies are usually not performed, however, patients tend to await elective management.

Postoperative infections leading to deep infective fluid collections were the most frequent cause of surgical readmission, accounting for 23% (n=3) of cases. These included two cases of subphrenic fluid collections and another case of an evolving abscess.

In one case, an individual in their 60s developed vomiting, right upper quadrant pain, and an elevated C-reactive protein (CRP) level on the first postoperative day. Computed tomography (CT) of the abdomen demonstrated a fluid collection in the gallbladder bed, associated perihepatic fluid, the presence of air locules, and a mildly dilated common bile duct. The patient responded well to antibiotic therapy.

A second patient, in their 20s, reported right upper quadrant discomfort in the early postoperative period. Abdominal ultrasound revealed a well-organized, largely anechoic fluid collection measuring approximately 2.9 × 1.9 cm. Conservative management with antibiotics led to the resolution of symptoms.

A third patient, in their 50s, presented to the emergency department two days postoperatively. CT imaging identified an extensive right-sided subphrenic fluid and gas collection measuring approximately 5 × 7 cm, with no radiological evidence of biliary obstruction. This patient was also managed successfully with antibiotic therapy.

Other reasons for readmission included abdominal pain n = 2 (15.4%), pancreatitis n = 2 (15.4%), bile leak n = 1 (7.7%), and nausea and vomiting n = 1 (7.7%). Our audit's findings align with other studies conducted in the United Kingdom and North America, where the most common readmissions were due to surgical causes [1].

Bile duct injury, which can lead to bile leaks, is one of the most serious complications of laparoscopic cholecystectomy [7]. In our audit, three patients experienced this complication: one was managed with endoscopic retrograde cholangiopancreatography (ERCP), while the other two required surgical intervention. Preoperative Magnetic resonance cholangiopancreatography (MRCP) has proven to be a valuable tool for evaluating biliary tract anatomy and identifying anatomical variants that may predispose patients to complications. Kang et al. emphasized the role of MRCP in patients with elevated γ-GT, recommending its use to mitigate risk [8]. In our audit, 61% of patients underwent preoperative MRCP or ERCP. Moreover, Halawani et al. analyzed the National Surgical Quality Improvement Program (NSQIP) database and reported a 15% reduction in readmission rates when intraoperative cholangiograms were performed [9]. In contrast, only eight patients in our study had an on-table intraoperative cholangiogram, highlighting a potential area for improving practice.

A patient in their 40s underwent an uneventful cholecystectomy. In the immediate postoperative period, the patient reported substernal, abdominal distension, and back pain accompanied by dyspnea. CT of the abdomen revealed free fluid around the liver, raising suspicion of a bile leak. A laparoscopic washout was performed. Following the procedure, the patient’s condition stabilized, with marked improvement in pain. Antibiotic therapy with gentamicin was initiated, and the remainder of the hospital stay was uneventful.

A patient in their 70s underwent an uncomplicated cholecystectomy but subsequently developed right upper quadrant abdominal pain associated with voluntary guarding. MRCP demonstrated a bile leak originating from the intrahepatic biliary system, specifically from the right anterolateral aspect of the common hepatic duct, with pooling of contrast in the gallbladder fossa. ERCP was subsequently performed, and a biliary stent was successfully inserted, resulting in clinical improvement.

A single mortality directly attributable to surgical complications was recorded. The patient, an elderly individual, developed right-sided abdominal pain, tachycardia, hypotension, and hypoxia on postoperative day nine. A CT scan of the abdomen and pelvis was performed to evaluate for intra-abdominal complications. Imaging revealed bilateral pleural effusions, a large gallbladder fossa hematoma extending into the perihepatic space, and a subcapsular liver hematoma. Active contrast extravasation (perivascular blush) was noted at the site of the previously ligated cystic artery, suggestive of arterial bleeding from the stump. A subsequent CT scan confirmed thrombosis of the right hepatic artery with evolving segmental infarctions in the right hepatic lobe. Despite US-guided drainage and angiographic embolization, the patient succumbed to complications related to hemorrhage and hepatic ischemia.

Limitations

Data collection was conducted retrospectively, focusing primarily on readmitted patients. This approach limited the exploration of potential risk factors, demographic information, and predictors for readmission.

Only 30-day readmission rates were analyzed, potentially overlooking cases where patients experienced delayed or long-term postoperative complications beyond the 30-day period.

Cholecystectomies performed in the context of malignancy are generally more complex than those for benign conditions, often requiring extended operative time, more extensive dissection, and careful management of altered anatomical planes. These procedures are associated with a higher risk of intraoperative and postoperative complications, including bile duct injury, bleeding, and infection. The increased morbidity is largely attributable to local tumor invasion, inflammation, or prior treatments such as chemotherapy or radiotherapy that distort normal tissue planes and vascular structures.

Most patients locally had an overnight hospital stay for observation as part of routine clinical practice, even for uncomplicated cholecystectomy. This practice may limit the comparability of results to other studies and possibly impact the 30-day readmission rate.

One notable limitation of this study is the absence of stratification based on the experience level of the operating surgeon. While laparoscopic cholecystectomy is known to have a learning curve, and operator expertise can influence complication rates, surgeries in our institution are often performed in a teaching environment involving collaborative efforts between trainees and supervising consultants. As such, attributing outcomes to a single operator level would introduce potential bias and confounding. Future studies specifically designed to evaluate surgeon experience as an independent variable may provide further insight into its role in postoperative outcomes.

One additional limitation of this study is the exclusion of surgical complications and readmissions that may have occurred at other local institutions. As the study focused solely on data collected from Mater Dei Hospital, complications managed outside of our institution were not captured, potentially limiting the comprehensiveness of the findings and their generalizability to the broader patient population.

Recommendations have been proposed. Given that the 30-day readmission rate following cholecystectomy at Mater Dei Hospital aligns well with Commission guidelines, we plan to promote same-day discharge for uncomplicated cases through informational posters and by presenting this audit at conferences. A prospective follow-up audit will be conducted to determine the impact of same-day discharge of uncomplicated laparoscopic cholecystectomies on the 30-day readmission rate.

## Conclusions

This audit concludes that the 30-day readmission rate following cholecystectomy is lower than 10%, in keeping with said target. Nonetheless, the complication rate was higher. Intraoperative cholangiogram may serve as a benefit in an effort to delineate complex biliary anatomy.
